# Rutin-Loaded Nanovesicles for Improved Stability and Enhanced Topical Efficacy of Natural Compound

**DOI:** 10.3390/jfb12040074

**Published:** 2021-12-13

**Authors:** Maria Chiara Cristiano, Antonella Barone, Antonia Mancuso, Daniele Torella, Donatella Paolino

**Affiliations:** 1 Department of Experimental and Clinical Medicine, University “Magna Græcia” of Catanzaro, 88100 Catanzaro, Italy; mchiara.cristiano@unicz.it (M.C.C.); barone@unicz.it (A.B.); dtorella@unicz.it (D.T.); 2 Department of Health Sciences, University “Magna Græcia” of Catanzaro, 88100 Catanzaro, Italy; antonia.mancuso@unicz.it

**Keywords:** nanovesicles, rutin, transcutaneous delivery, antioxidant activity

## Abstract

Rutin is a natural compound with several pharmacological effects. Among these, antioxidant activity is one of the best known. Despite its numerous benefits, its topical application is severely limited by its physicochemical properties. For this reason, the use of suitable systems could be necessary to improve its delivery through skin, thus enhancing its pharmacological effects. In this regard, the aim of this work is to optimize the ethosomal dispersion modifying both lipid and ethanol concentrations and encapsulating different amounts of rutin. Characterization studies performed on the realized systems highlighted their great stability properties. Studies of encapsulation efficiency and loading degree allowed us to identify a better formulation (EE% 67.5 ± 5.2%, DL% 27 ± 1.7%), which was used for further analyses. The data recorded from in vitro studies showed that the encapsulation into these nanosystems allowed us to overcome the photosensitivity limitation of rutin. Indeed, a markable photostability of the loaded formulation was recorded, compared with that reported from the free rutin solution. The efficacy of the nanosystems was finally evaluated both in vitro on keratinocyte cells and in vivo on human healthy volunteers. The results confirmed the potentiality of rutin-loaded nanosystems for skin disease, mainly related to their anti-inflammatory and antioxidant effects.

## 1. Introduction

From ancient times, the application of natural products was widespread as therapy for several pathological conditions [1] such as skin inflammation, erythematous rashes, skin photosensitivity, urticaria, psoriasis, etc. Currently, alongside the nanomedicines’ development, the transcutaneous administration route was deeply investigated and improved [2] in order to ameliorate the molecule’s efficacy.

The first limiting step for a transcutaneous route is surely the barrier effect of stratum corneum epidermis (SCE), and for this reason, several research teams focused their attention on even new approaches to bypass this drawback and on the performance of micro- and nanosystems such as nanoemulsions, deformable nanovesicles, solid lipid nanoparticles, and nanostructured lipid carriers and micelles [3].

Among deformable nanovesicles, ethosomes^®^ have represented one of the main biocompatible nanosystems that better cross the skin layers, thus providing an effective and safe drug delivery [4]. This need arises from the limitations of conventional nanosystems such as liposomes because of their lack of deformability, leading to a massive release of cargo in the upper SCE [5]. On the contrary, ethosomes’ composition and in particular the presence of ethanol in the phospholipid bilayer as a penetration enhancer to increase its deformable capability permit the deeper layers to be reached both in an occlusive and in a non-occlusive manner [6]. Since they were conceived [7], these nanocarriers were performed for the topical delivery of several compounds, both hydrophilic and lipophilic ones [8]. In this regard, Godin et al. demonstrated that ethosomes can exploit the intercellular lipid domains between corneocytes in the SCE, thus allowing for drug penetration [9].

Skin disorders affect most of the world’s population, and a part of these, such as atopic dermatitis, can be associated with the high presence of reactive oxygen and nitrogen species (ROS and RNS), leading to an acute exacerbation of the pathologies [10,11]. This altered homeostasis sometimes results in irreversible states such as lipid and protein oxidation, and nucleic acid damages [12]. For these reasons, the involvement of free radical scavengers is crucial to obtaining a cytoprotective function capable of preventing oxidative stress by quenching free radicals [13].

In this respect, rutin is a natural compound, known also as rutoside, with several pharmacological purposes, from anti-inflammatory to cardioprotective ones, playing also an important role in the oxidant–antioxidant balance associated with some diseases [14,15]. This compound leads to a higher secretion of glutathione, of which the function is to detoxify hydrogen peroxide to water [16]. Furthermore, rutin affects oxidative stress by inhibiting xanthine oxidase, of which activation leads to uric acid production and superoxide radicals’ formation [17,18].

The aim of the work is to provide a comparative physicochemical analysis of ethosomal nanocarriers in order to optimize and choose the proper nanosystem composition better suited for topical rutin delivery, exploiting its broad spectrum of activity. Each formulation was properly analyzed in terms of dimensional values, stability parameters, deformability features, entrapment efficiency, and loading degree. Moreover, after this thorough physicochemical characterization, the efficacy of the resulting better rutin-loaded formulation was investigated in vitro and then in vivo on healthy human subjects, confirming that the loaded and optimized systems improve the antioxidant profile of the natural compound.

## 2. Materials and Methods

### 2.1. Materials

Soy phosphatidylcholine (purity ≥ 93 ± 3%, or Phospholipon 90^®^ G) was obtained from Natterman Phospholipid GMBH, Cologne, Germany. Absolute ethanol (European Pharmacopeia, analytical reagent) was purchased from Carlo Erba (Milan, Italy). Rutin was furnished by Sigma-Aldrich (Sigma Aldrich GmbH, Steiheim, Germany). Methyl-nicotinate (purity > 99%) was a Sigma–Aldrich product (Germany). The Instituto Zooprofilattico of Modena and Reggio Emilia (Reggio Emilia, Italy) provided the NCTC 2544 cell line. Dulbecco’s modified Eagle medium with glutamine, trypsin/ethylenediaminoacetic acid (EDTA) solution, fetal bovine serum (10% *v*/*v*), penicillin-streptomycin solution (1% *v*/*v*), and 3-[4,5-dimethylthiazol-2-yl]-3,5-diphenyltetrazolium bromide salts were furnished by Invitrogen (Life Technologies, Milan, Italy). All other chemicals were of analytical or HPLC grade. Double-distilled water was used for the study.

### 2.2. Methods

#### 2.2.1. Ethosomes^®^ Preparation

Colloidal dispersion was prepared as Touitou et al. previously described [19], with slight modification. Briefly, 1% and 2% (*w*/*v*) of Phospholipon 90^®^ G was solubilized with a suitable amount of ethanol in order to obtain a final concentration of 30% and 40% (*w*/*v*) into Pyrex^®^ glass vials. Double distilled water was slowly added under continuous mixing at 700 rpm with a magnetic stirrer (Midi MR1 Digital Ikamag^®^; IKA-WERKE GMBH and Co., Staufen, Germany), preventing ethanol evaporation through an airtight cap connected to a syringe system with a Teflon tube. The resulting formulation was homogenized at 15,000 rpm for 1 min using Ultra-Turrax T 25 equipped with an S25N-8G homogenizing probe (IKA-WERKE). Finally, the milky dispersion was left at room temperature for 30 min under continuous stirring (Orbital Shaker KS 130 Control, IKA-WERKE) and then filtered at 0.22 µm [20].

Rutin was initially added to ethanol in order to permit its solubilization at different amounts, obtaining the following final concentrations: 0.5 mg/mL, 1 mg/mL, 2 mg/mL, 3 mg/mL and 4 mg/mL (Table 1). Due to the photosensitivity of rutin, the preparation method were carried out while keeping rutin and the obtained preparations away from light.

#### 2.2.2. Physicochemical Characterization of Ethosomes^®^

Colloidal dispersions were characterized in terms of particle size, polydispersity index (PdI), and zeta potential (ZP) using a Zetasizer Nano ZS (Malvern Instruments Ltd., Worcestershire, United Kingdom). In particular, the size and PdI were fitted to the third-order cumulant expression and at a back scattering angle of 173° [21,22], while zeta potential is related to electrophoretic mobility using Smoluchowsky equations [23]. The samples were diluted with a proper aqueous solution to avoid multi-scattering phenomena.

All measurements are the mean of three different experiments ± standard deviation.

#### 2.2.3. Deformability Test

All ethosomes^®^ dispersions, empty and loaded, were analyzed in terms of deformability features by extrusion through proper pore size polycarbonate membrane filters, chosen to ensure that pore dimensions were 1/3 compared with particle sizes [24] at a constant pressure of 2.5 bar for 10 min using a Lipex Biomembranes extruder (Northern Lipids Inc., Vancouver, BC, Canada). The elasticity of vesicles was reported as a deformability index (***DI***) (Table 2), according to the following equation [25]:

(1) $$DI=\left[J\left(\frac{d_{0}}{p}\right)\left(\frac{d_{0}}{d_{0}-d_{1}}\right)\right]$$

where ***J*** is the ratio between the weight of ethosomes^®^ before and after extrusion, ***d*_0_** and ***d*_1_** are the average diameters of nanocarriers before and after extrusion, and ***p*** is the pore size of polycarbonate membrane filters. The results are expressed as the average of three different experiments ± standard deviation. Conventional liposomes were used as a negative control [26].

#### 2.2.4. Rutin Entrapment Efficiency and Loading Degree

The contents of the encapsulated rutin in ethosomes^®^ was determined through spectrophotometric analyses. In particular, ethosome’s dispersion was centrifuged using Avanti 30 Centrifuge (Beckman, Fullerton, CA, USA) equipped with a fixed angle rotor Beckman mod. F1202 at 56,000× *g* for 1 h at 4 °C [20]. The resulting supernatant was separated and analyzed using the Perkin Elmer Lambda 35 UV-Vis spectrophotometer (Waltham, Massachusetts, USA) at a wavelength of 359 nm. The entrapment efficiency (***EE%***) and drug loading degree (***DL%***) were calculated according to the following equations:

(2) $$EE\%=\left[\frac{D_{t}-\;D_{s}}{D_{t}}\right]*100$$

(3) $$DL\%=\left[\left(D_{t}-D_{s}\right)/L_{w}\right]*100$$

where ***D_t_*** is the total amount of rutin used during the preparation phase, ***D_S_*** is the amount of rutin recovered in the supernatant, and ***L_w_*** is the amount of lipid used.

#### 2.2.5. Stability Analysis of Ethosomes^®^

Turbiscan Lab^®^ Expert stability analysis (Formulaction, L’Union, France) was used to predict the long-term stability of the formulations through the variations of light transmitted (ΔT) and backscattered (ΔBS) at 180° and 45° with respect to the incident laser and then received by two different synchronous optical sensors. Furthermore, turbiscan stability index (TSI) values were provided from this investigation in order to identify the destabilization kinetic profiles of ethosomal formulations [27]. Each sample was properly diluted to 1:10 (final volume 10 mL) with aqueous solution and placed into a cylindrical glass tube before being analyzed at 25 °C for 1 h.

#### 2.2.6. Photostability Test

The resulting optimized rutin-loaded ethosomes formulation (NV-A_4_) and an ethanol solution of rutin (both at 30 µg/mL) was placed into 1 × 1 cm quartz cuvette, and continuously irradiated with a Xenon lamp. The used wavelength range was from 300 to 800 nm, according to the ICH Guideline. The rutin absorbance (359 nm) was recorded at different time points of 0, 15, 30, 45, 60, 90, and 120 min using a UV-Vis spectrophotometer (Perkin Elmer Lambda 35, Waltham, MA, USA) [28].

#### 2.2.7. In Vitro Cellular Studies

The in vitro cytotoxic activity of rutin-loaded ethosomes (NV-A_4_) was evaluated on keratinocyte cells (NCTC2544) using an MTT assay, and the results were expressed in terms of cell viability. The cells were seeded at a density of 5 × 10^3^ cells/well in 96-well plates. After 24 h of incubation, the cells were treated with 100 µL of the fresh medium added with empty ethosomes^®^ or DMSO, used as negative controls, at several concentrations referred to loaded ethosomes^®^ and free active solution tested during the evaluation of antioxidant effect. Hence, 10 µL of MTT solution (5 mg/mL dissolved in PBS solution) was added to each well. After 3 h of incubation, precipitated formazan salts were dissolved with 100 µL of ethanol–DMSO solution (1:1 *v*/*v*), and the plate was shaken for 20 min at 230 rpm (IKA^®^ KS 130 Control, IKA^®^ WERKE GMBH & Co., Staufen, Germany).

The cell viability (%) was determined by the use of microplate spectrophotometer (Multiskan MS 6.0, Labsystems, Vantaa, Finland) at a wavelength range between 540 nm and 690 nm using the following equation:

(4) $$Cell\;viability\;\left(\%\right)=\left(\frac{Abs_{t}}{Abs_{c}}\right)*100$$

where ***Abs_t_*** represents the absorbance of treated cells and ***Abs_c_*** represents the absorbance of untreated ones.

For antioxidant efficacy, NCTC 2544 cells were seeded in a 96-well plate at a density of 7.5 × 10^3^ cells/well. After 24 h of incubation, the cells were treated with free active solution or with rutin-loaded ethosomes (NV-A_4_) at the concentrations of 0.1, 1, 10, 50, and 100 µM. After 24 h of treatments, each well was treated with 700 µM of H_2_O_2_ for 1 h, and then the MTT assay was used to obtain cell viability values in order to investigate the cytoprotective effect of rutin. The results were reported as the mean value of three independent experiments ± standard deviation.

#### 2.2.8. In Vivo Anti-Inflammatory Effect on Human Volunteers

In order to investigate its potential anti-inflammatory effect, rutin-loaded ethosomes^®^ (NV-A_4_) were investigated compared with NaCl 0.9% (*w*/*v*) aqueous solution and a water–ethanol solution of rutin. The analysis was performed on 10 human healthy volunteers (mean age, 25 ± 5 years; both sexes) under controlled experimental conditions of temperature and humidity (25 °C and 40%). Briefly, three sites for each human volunteer forearm of about 1 cm^2^ were previously analyzed for baseline values using a reflectance visible spectrophotometer, SP60 (X-Rite Incorporated). Then, each site was pre-treated with 200 μL of methylnicotinate solution (0.2% *w*/*v*), used as an irritative agent, for 15 min to chemically induce an inflammation. After this pre-treatment, 200 μL of saline solution (NaCl 0.9% *w*/*v*), rutin hydroalcoholic solution (H_2_O:EtOH 70:30 *v*/*v*), and rutin-loaded ethosomes^®^ were applied in non-occlusive conditions. Both rutin-loaded ethosomes^®^ and the free rutin solution used had concentrations of 2.7 mg/mL.

The induced erythema (***EI***) was calculated using the following equation [29]:

(5) $$EI=100\left[loglog\;1/R_{560}\;+1.5\left(loglog\;\frac{1}{R_{540}}+loglog\;1/R_{580}\right)-2\left(loglog\;\frac{1}{R_{510}}+loglog\;1/R_{610}\right)\;\right]\;$$

where ***R*** is the reflectance at specific wavelengths (510, 540, 560, 580, and 610 nm). The spectrophotometric analysis was carried out at 1 h, 2 h, 3 h, 4 h, and 5 h and the results are reported as Δ***EI*** (%) ± standard deviation.

#### 2.2.9. Statistical Analysis

Statistical significance was carried out using a one-way ANOVA test. A posteriori Bonferroni t-test was carried out to check the ANOVA test. The level of significance was set at * *p* < 0.05 and ** *p* < 0.001. All data are shown as mean value ± standard deviation.

## 3. Results and Discussion

### 3.1. Physicochemical Features of Ethanolic Nanovesicles

Physicochemical features of active molecules, such as molecular weight, partition coefficient, etc., strongly affect their cutaneous applicability [30]. Indeed, unsuitable parameters may hamper or reduce molecules’ ability to cross the stratum corneum epidermis (SCE), thus limiting their efficacy [31].

In this scenario, several nanocarriers, such as ethosomes^®^ and transfersomes^®^, have been designed in order to reduce these drawbacks and to improve the accumulation of payloads into deeper skin layers [32]. In particular, an effective nanosystem for topical application should cross the SCE intact, allowing for a proper accumulation of cargos in pathological tissues. For these reasons, the physicochemical characteristics of drug delivery systems play pivotal roles and their optimization during the early stages of study is crucial to develop an effective nanomedicine [33].

In these attempts, several ethosomal formulations characterized by different qualitative and quantitative compositions were prepared and the average diameter, size distribution, and zeta potential of all realized nanocarriers were evaluated by using DLS technique (Table 2).

As Table 2 shows, the loading of rutin into ethosomes^®^ strongly influences the physicochemical characteristics of the resulting therapeutic nanosystems, leading to certain differences compared with empty ones. Except for NV-D_4_, therapeutic nanovesicles demonstrated a progressive reduction in the average diameter by improving the amount of rutin loaded during the preparation phases. This phenomenon was not totally clear, but probably, the active compound may act as packaging agent, thus balancing/reducing the fluidizing effect of ethanol and thus leading to a more rigid bilayer structure. Our hypothesis is also confirmed by the deformability index (D.I.) values, which were found proportionally lower by improving the rutin amount. Probably, because of its chemical structure, rutin can interact with both the acyl chains and polar heads of phospholipids with aromatic rings and hydroxyl groups, respectively. Despite this reduction, the D.I. values can be considered suitable enough for a potential cutaneous applicability, also compared with the deformability feature of liposomes, considered as rigid and no-elastic vesicles [24]. The packaging effect of the active molecule was higher in the C and D sample series. Indeed, despite the samples C and D showing higher D.I. compared with A and B in response to the improved ethanol percentage, respectively. the deformability of all samples realized by using 4 mg/mL of rutin (NV-A_4_, NV-B_4_, NV-C_4_, and NV-D_4_) was very similar (Table 2).

Moreover, the zeta potential was investigated in order to ameliorate the formulation design because the presence of a net surface charge (positive or negative) may improve the colloidal stability of nanodispersions through an electrostatic repulsion among the nanovesicles [34]. A slight reduction in zeta potential values (ranging between 2 and 6 mV) was observed for all samples in response to adding rutin during the preparation stages. These changes were probably due to the physical partition of the active that may interact with bilayer phospholipids’ groups, thus slightly modifying the final zeta potential. Despite this, the zeta potential values of rutin-loaded ethosomes^®^ were sufficiently negative to provide a suitable colloidal stability, except for the samples of D series. Indeed, despite the NV-D (empty nanovesicles made up of 2% *w/v* of PL90G^®^ and 40% *w/v* of ethanol) in agreement with our previous results [24] demonstrating the lowest zeta potential value (−40.3 mV ± 1.6), the presence of rutin strongly reduces this parameter, suggesting a potential colloidal instability. This trend was similar for all samples of D series, except for NV-D_0.5_, which showed a zeta potential of −33.6 mV ± 1.70. Conversely, a huge increment in average size compared with the relative empty formulation (603 nm ± 10 vs. 204.1 nm ± 2.3) for NV-D_0.5_, demonstrating destabilization phenomena. For the abovementioned reasons, the samples of D series were excluded from the studies.

Furthermore, the NV-A_1_, NV-A_2_, NV-A_3_, and NV-C_4_ showed PdI values that exceeded the threshold of 0.3, thus suggesting a not homogenous size distribution [35]. For this reason, these samples were also withdrawn from the studies. It is noteworthy that the formulations of A series demonstrated increased PdI when the amount of active greater than 0.5 mg/mL was used, while this value dropped below 0.2 for NV-A_4_ (0.16 ± 0.014). This behavior was not completely understood, but probably, this lipid:rutin ratio leads to stabilization of the formulation, becoming more stable than the relatively empty one.

### 3.2. Rutin Entrapment Efficiency and Loading Degree

Other important parameters taken into account during this investigation were the rutin entrapment efficiency (EE%) and the active loading degree (DL%) of resulting nanovesicles, indicating the quality of the formulations [36]. The EE% and the DL% were evaluated for the formulations not discarded during the previous analysis using an appropriate external calibration curve (Figure S1).

**Figure 1:**
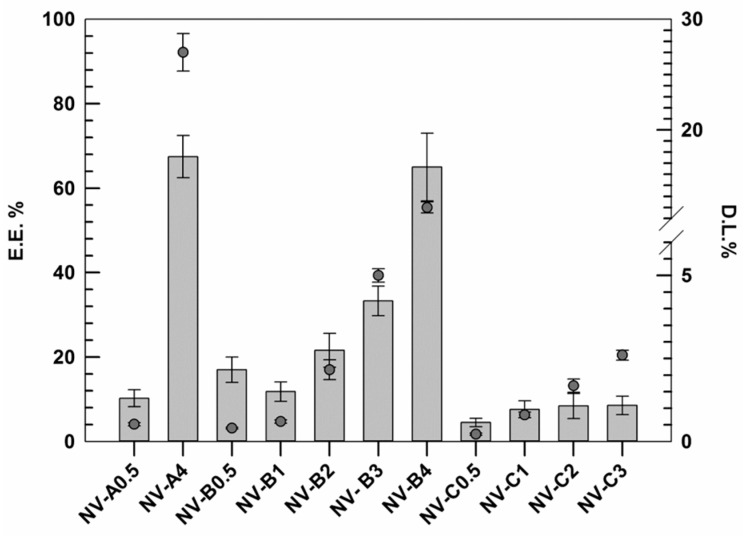
Rutin entrapment efficiency (E.E.%) and loading degree (D.L.%) of several formulations. The results are expressed as the mean value of three independent experiments ± standard deviation.

The results demonstrated that the EE% of rutin, as well as the DL% increased by improving the amount of rutin used during the realization stages (Figure 1).

This trend was similar for all investigated formulations (except NV-B_0.5_), and probably, it was due to the presence of ethanol. In this context, the ethanol may act as co-solvent, thus improving the solubility of hydrophobic active molecules. In these attempts, probably, the low amount of active was massively removed during the purification step in response to the well-known ethanol fluidifying effect on lipid membrane and the improved solubility in the medium [37]. Conversely, by improving the active amount, the nanovesicles membrane showed a proportionally improved rigidity, as discussed above. This finding, associated with a progressive reduction in the ethanol:rutin ratio, may justify this behavior. Our hypothesis is also confirmed by results obtained with samples C series. In fact, in these formulations made using higher amounts of ethanol (40% *w/v* instead of 30% *w*/*v*), the increment in EE% always resulted lower than those in the A and B series.

Based on reported data, NV-A_4_ and NV-B_4_ showed the best results in terms of EE%, 67.5 ± 5.2 and 65.2 ± 8.3, respectively. However, despite these values being very similar and no significant differences being found between each other, NV-A_4_ showed a D.L.% twice as high as NV-B_4_ due to the lower amount of lipid used (27 ± 1.7% vs. 13 ± 0.5%, respectively). For this reason, in addition to the suitable physicochemical properties shown above, NV-A_4_ was selected as the best one and used for further investigations.

### 3.3. Stability Evaluation through Turbiscan Lab Expert Analysis

One of the main challenges in the field of nanomedicine is to realize a drug delivery system with long-term stability in order to make its production advantageous on a large scale, thus enhancing the real applicability in the clinic via the translation from bench to bedside [38]. In order to confirm the physical stability of the selected formulation, turbiscan analyses were carried out for both empty and rutin-loaded ethosomes^®^, NV-A and NV-A_4_, respectively. Turbiscan analysis is a non-disruptive technique that allows a user to predict the long-term stability of nanocarriers by investigating potential physical instability phenomena, i.e., flocculation, sedimentation, or creaming, of colloidal dispersions [39]. The two parameters taken into account during this analysis are delta Back Scattering (ΔBS%) and delta Transmittance (ΔT%). Indeed, variations in these parameters below 5% demonstrate the absence of the destability phenomena described above [25].

**Figure 2:**
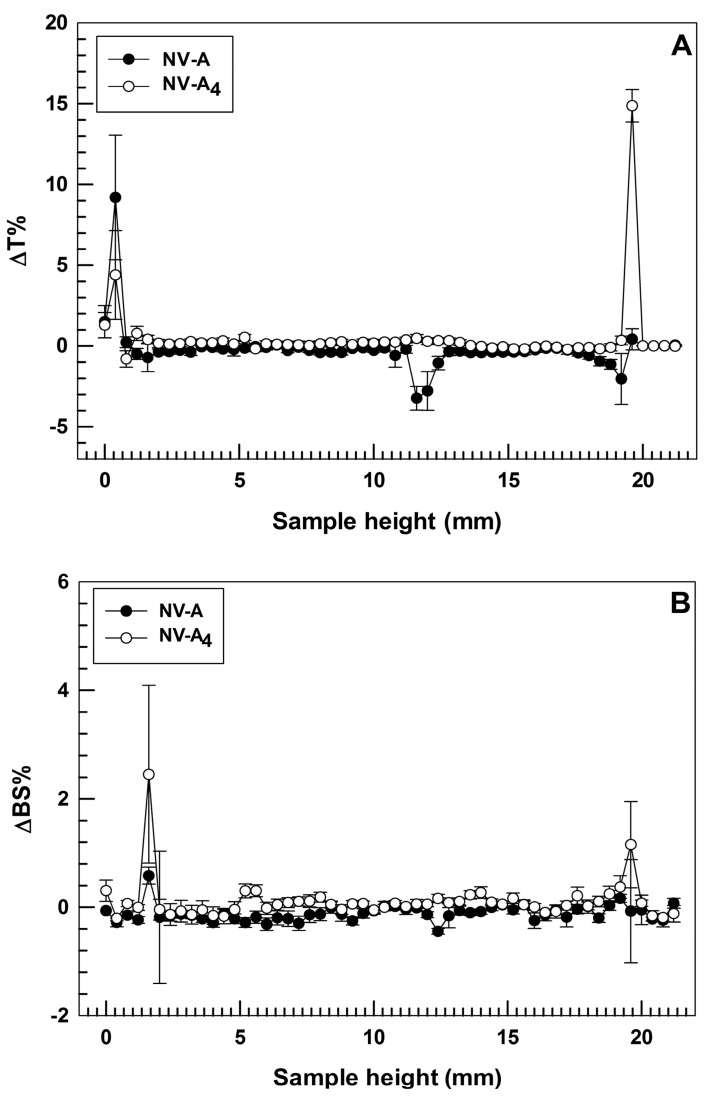
Stability studies performed using Turbiscan Lab^®^ Expert. Data are expressed as (**A**) delta-transmission (ΔT) and (**B**) delta-backscattering (ΔBS) profiles recorded from formulation NV-A and NV-A_4_. Each value is reported as a function of time (1 h) and sample height.

In these attempts, the two investigated formulations demonstrated ΔT% and ΔBS% below the threshold value for the entire sample’ s height during the analysis at room temperature, showing almost overlapping profiles (Figure 2). This finding is in agreement with our hypothesis related to the zeta potential value and confirms that the loading of rutin into ethosomes^®^ did not compromise their physical stability. It is also noteworthy that NV-A showed slight variations in ΔT profile (even below than 5%) between 11 and 13 mm of the sample height (Figure 2A). Meanwhile, NV-A_4_ did not show this variation, confirming the previous hypothesis about the stabilization activity of rutin on the ethosomes’ structure. The only significant variations in ΔT profiles were observed at ca. 2 mm and ca. 20 mm of the sample height, even if this is not related to the occurrence of destabilization phenomena but probably because of the air bubbles in the glass vial at the top and bottom interfaces.

**Figure 3:**
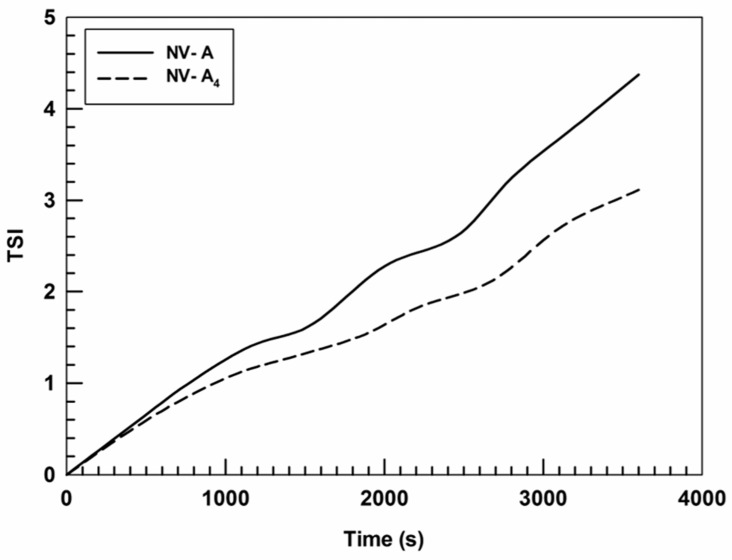
Kinetic stability (TSI) profiles of NV-A and NV-A_4_. Panel reports representative data from three independent experiments. Data are reported as a function of time (1 h) and sample height.

Moreover, the turbiscan stability index (TSI) confirmed the data obtained by the formulation profiles of ΔT% and ΔBS%, showing that the insertion of rutin in the nanostructure leads to an A_4_ TSI value lower than that of NV-A (Figure 3).

### 3.4. UV-Photostability Studies

The antioxidant features of rutin are well known from the past few years and were exploited for several purposes [40,41]. Its mechanism is due to the structure of this molecule and to the different conjugated aromatic rings that provide radical scavenger properties [42]. However, the applicability of the rutin results are limited in response to its light sensitivity, which modifies the active structure, thus leading to a reduced antioxidant effect.

**Figure 4:**
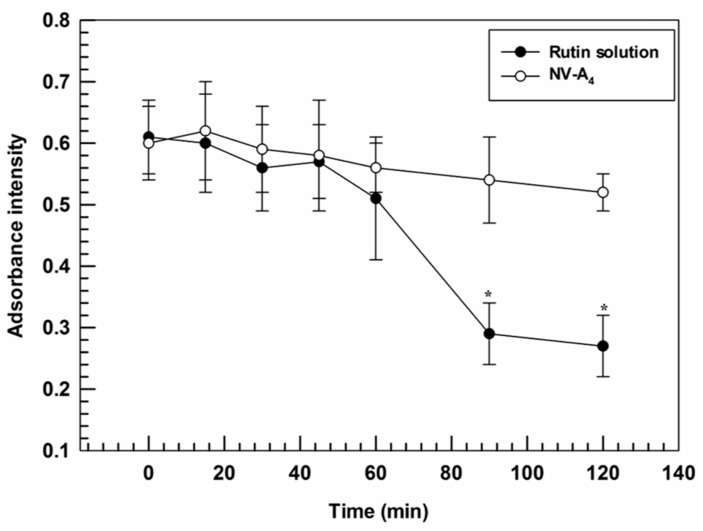
Degradation profile of NV-A_4_ and the free rutin solution as a function of UV-exposure time. The results are expressed as mean values of three independent experiments ± standard deviation. * *p* < 0.05 of rutin solution compared with NV-A_4_.

Furthermore, the ability of ethosomes^®^ to improve the photostability of loaded actives was investigated. Indeed, a suitable nanosystem should be able to both promote the accumulation of cargo in the target tissues and to protect the payloads from the physical or chemical degradation [43]. In line with this required property, ethosomes^®^ were demonstrated to provide a suitable protection against photo-degradation induced by continuous exposure to UV-light, thus acting as photostabilizers. Significant differences were found between NV-A_4_ and the free rutin solution after 90 min of UV-exposure, showing absorbance intensities of 0.54 ± 0.07 and 0.29 ± 0.05, respectively. A slight hypochromic effect was also observed for NV-A_4_ after 120 min, from 0.61 ± 0.06 to 0.52 ± 0.03, but this decrease was not significant (Figure 4). These findings highlight the ability of ethosomes^®^ to reduce the UV-induced degradation rate of rutin, a key factor in a potential topical application.

### 3.5. In Vitro Cell Study Evaluation

**Figure 5:**
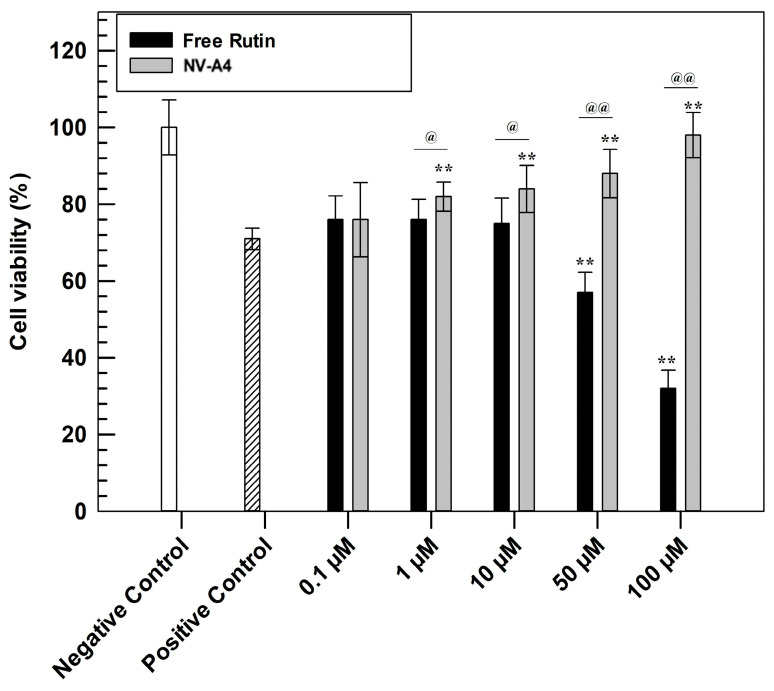
In vitro antioxidant efficacy of NV-A_4_ vs. free rutin solution. Statistical differences were analyzed between treatments and positive control (*) and between free active solution and rutin-loaded ethosomes^®^ (@). The results are expressed as mean values of three independent experiments ± standard deviation.

The evaluation of antioxidant features was carried out to confirm the efficacy of the designed loaded formulation. This investigation was performed through in vitro studies on the NCTC 2544 cell line using MTT tests (Figure 5). Briefly, to confirm the cytoprotective effect, a pre-treatment of 24 h with increasing concentrations of NV-A_4_ and rutin in free form was carried out on the NCTC 2544 cell line. Subsequently, oxidative stress conditions were mimicked by adding hydrogen peroxide (700 µM) for 1 h, leading to the formation and further accumulation of ROS [44]. After 1 h of induced stress, the MTT salts were metabolized into formazan crystals by living cells’ mitochondria. Finally, after crystals dissolution, the measured absorbances allow us to obtain a percentage of cell viability.

Observing Figure 5, the higher antioxidant effect of rutin-loaded ethosomes, compared with rutin in free form, was immediately evident. In detail, the protective effect of NV-A_4_ was significantly shown already at the concentration of 10 µM, leading the highest tested concentration to a similar cell viability percentage of untreated cells (negative control). Conversely, the free active solution shows results comparable with the positive control (cells treated with H_2_O_2_ in absence of rutin) up to 10 µM, not providing a sufficient cytoprotective action. Furthermore, ethosomes can provide the cellular penetration of antioxidant compounds [45]. On the contrary, free rutin solution at concentrations of 50 and 100 µM showed even lower cell viability percentages compared with the positive control; this accentuated cytotoxic effect of rutin in free form was due to the DMSO (dimethyl sulfoxide) amount, used as solvent for its solubilization. To confirm this hypothesis, another MTT assay was carried out on NCTC 2544, testing increasing concentrations of DMSO and evaluating the safety profile of empty ethosomes, using the amount of empty carriers necessary to reach the rutin-loaded ethosomes concentration previously tested in an antioxidant test. As reported in Figure S2, empty NV-A showed a safe and biocompatible profile at all tested concentrations, maintaining a cell viability higher than 80% up to 72 h of treatments. This result confirmed that these nanocarriers can interact with cells without resulting in damaging subcellular alterations. On the other hand, the MTT results, obtained after the cell treatment with increasing DMSO concentrations, confirmed that the solvent induced a significant reduction in NCTC 2544 cell viability already after 24 h at the higher tested concentration.

The obvious advantage that is derived from the use of nanotechnology explains how to overcome the dose-dependent cytotoxic effect of actives or the solvent to solubilize it, thus ensuring better benefits but reducing dosages’ disadvantages.

### 3.6. In Vivo Efficacy on Human Volunteers

**Figure 6:**
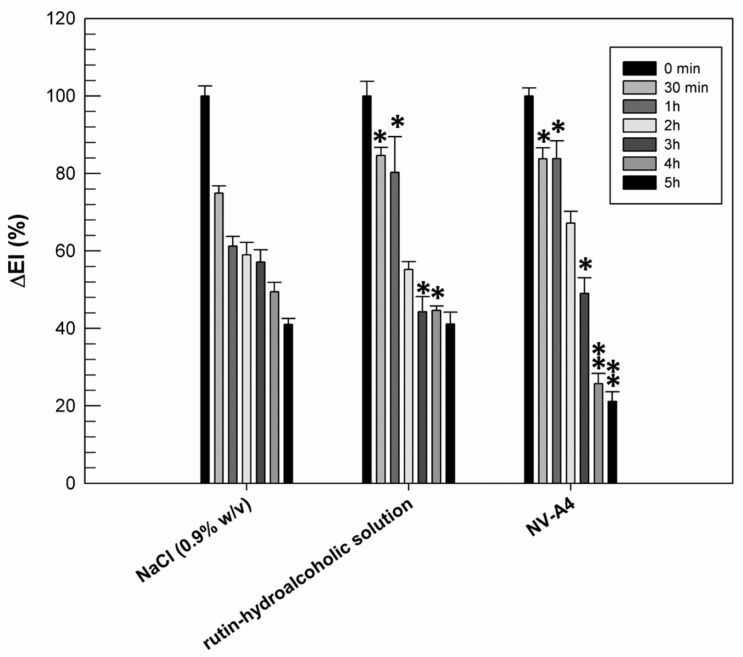
Erythema index variation (ΔEI%) measured after topical application of the NaCl solution (0.9% *w*/*v*), the rutin–hydroalcoholic solution, and rutin-loaded ethosomes (NV-A_4_), on inflamed forearms of human healthy volunteers. The results were expressed as mean values ± standard deviation. * *p* < 0.05 and ** *p* < 0.001 compared with NaCl (0.9% *w*/*v*), used as a control.

The previously reported in vitro results confirmed the improved antioxidant activity of rutin when it is encapsulated within ethosomes. Another noteworthy pharmacological activity of rutin is its anti-inflammatory one, resulting from the scavenging ROS and the reduction in pro-inflammatory cytokines [46]. Based on this feature, we wanted to evaluate if the entrapment of rutin within ethosomal formulation (NV-A_4_) could improve the ability of natural compounds to make a reduction in chemically induced erythema on human volunteers. For this purpose, rutin-loaded ethosomes (NV-A_4_) and a hydroalcoholic solution of the natural compound were applied on the forearm of human healthy volunteers, previously inflamed using an irritative solution of methylnicotinate. The reduction in chemically induced erythema was then recorded and are reported in Figure 6.

From Figure 6, we can observe the physiological restoration of skin integrity and the reduction in inflammation in terms of erythema index, as a function of time, when a solution of NaCl (0.9% *w*/*v*) was used as the control. Observing the effect of rutin applied as a hydroalcoholic solution, we can note that, during the first 30 min and in the first hour of experiment, the induced inflammation was maintained at significantly higher values (*p* < 0.05) with respect to the same sites treated with NaCl. Probably, the persistence of the inflammation at these experimental times was due to the irritating effect of the ethanol, used to better solubilize and apply the rutin. On the contrary, after 5 h of treatment, the reduction in inflammation induced by the hydroalcoholic solution of rutin was similar to the physiological restoration of the skin (41.14 ± 3.05% for rutin hydroalcoholic solution and 41.02 ± 1.55% for NaCl). This result was probably due to a counterbalance between the irritating effect of ethanol and the anti-inflammatory effect of rutin. About rutin-loaded ethosomes, the beneficial effect of encapsulated rutin was already shown after 3 h of experiment, when a significant reduction in chemically induced inflammation was recorded with respect to NaCl treatments. Moreover, the anti-inflammatory effect of rutin-loaded ethosomes was more evident after 4 and 5 h of experiments: ethosomes induced a significant reduction (*p* < 0.001) in inflammation when compared with NaCl and rutin in free form. This result is very encouraging because it confirmed the ability of ethosomes to interact effectively with the lipid structures of the skin, to cross the stratum corneum, and to subsequently release rutin, exerting its anti-inflammatory pharmacological activity. Obviously, a certain time interval is necessary to obtain this effect, and for this reason, this benefit is not immediately evident.

## 4. Conclusions

The results obtained from this research work confirmed the suitability of ethosomes as topical drug delivery systems for a pharmacologically active natural compound, namely rutin. An in-depth physicochemical characterization allowed us to select the formulation NV-A_4_ as the most suitable among the prepared ethosomes. In fact, thanks to the qualitative and quantitative compositions of NV-A_4_, it showed suitable properties for topical administration and the best encapsulation efficiency values and drug loading degree of rutin. The presence of rutin in the formulation does not cause any relevant change in the features of nanovesicles, which retained their suitability for topical administration. However, rutin seemed to act as a packing agent, intercalating into the bilayer and causing a strengthening of the structure, although they retained good deformable properties. The results recorded from further in vitro studies showed that rutin-loaded nanocarriers protect cells against oxidation, showing the best anti-oxidant efficacy compared with the effect recorded from the free active solution. The photoprotective activity of rutin-loaded formulations was also recorded during in vitro UV-photostability studies. Indeed, ethosomes were able to protect rutin from a photodegradation process when it was exposed to constant UV light stress.

Finally, in vivo studies performed on human volunteers showed the anti-inflammatory efficacy of rutin-loaded ethosomes, particularly after 4 h and 5 h following an inflammatory process.

Of course, it is well known that in vitro studies carried out on only one cell line (NCTC 2544 cell line) can appear limited when ensuring the total absence of cytotoxicity of nanosystems, but thanks to in vivo studies, ethosomes showed not only their ability to improve the anti-inflammatory activity of entrapped rutin but also their safe profile on human volunteers. Finally, our results showed that the optimization of ethanolic nanovesicles as topical delivery systems for rutin permitted us to define a potential efficacious phytonanomedicine, of which the transcutaneous use could counteract the effects of several skin diseases. In detail, the antioxidant and anti-inflammatory effects of rutin were greatly enhanced from its encapsulation in the realized deformable nanovesicles.

## Figures and Tables

**Table 1:** Qualitative and quantitative compositions of various ethosomal formulations.

Formulation ^1^	EtOH (% *w*/*v*)	PL90G^®^ (% *w*/*v*)	H_2_O (% *w*/*v*)	Rutin (mg/mL)
NV-A	30	1	69	-
NV-A_0.5_	30	1	69	0.5
NV-A_1_	30	1	69	1
NV-A_2_	30	1	69	2
NV-A_3_	30	1	69	3
NV-A_4_	30	1	69	4
NV-B	30	2	68	-
NV-B_0.5_	30	2	68	0.5
NV-B_1_	30	2	68	1
NV-B_2_	30	2	68	2
NV-B_3_	30	2	68	3
NV-B_4_	30	2	68	4
NV-C	40	1	59	-
NV-C_0.5_	40	1	59	0.5
NV-C_1_	40	1	59	1
NV-C_2_	40	1	59	2
NV-C_3_	40	1	59	3
NV-C_4_	40	1	59	4
NV-D	40	2	58	-
NV-D_0.5_	40	2	58	0.5
NV-D_1_	40	2	58	1
NV-D_2_	40	2	58	2
NV-D_3_	40	2	58	3
NV-D_4_	40	2	58	4

^1^ NV = Nanovesicles; the numbers in subscript within the formulations name correspond to the concentrations of rutin used for their preparation.

**Table 2:** Physicochemical properties of nanocarriers.

SAMPLE	Size (nm)	PdI ^1^	ZP ^2^ (mV)	D.I. ^3^
NV-A	118 ± 1	0.237 ± 0.003	−28.6 ± 1.5	5.4 ± 1.0
NV-A_0.5_	299 ± 15	0.104 ± 0.031	−26.5 ± 1.3	5.2 ± 1.8
NV-A_1_	286 ± 14	0.363 ± 0.014	−25.2 ± 0.2	4.1 ± 1.6
NV-A_2_	246 ± 14	0.303 ± 0.060	−24.6 ± 1.7	3.8 ± 1.5
NV-A_3_	229 ± 13	0.351 ± 0.027	−25 ± 1.6	3.7 ± 1.6
NV-A_4_	112 ± 2	0.160 ± 0.014	−25.7 ± 1.3	3.5 ± 0.5
NV-B	136 ± 1	0.135 ± 0.032	−29.1 ± 2.2	7.1 ± 1.1
NV-B_0.5_	201 ± 1	0.210 ± 0.013	−27.6 ± 2.2	6.5 ± 1.3
NV-B_1_	162 ± 2	0.170 ± 0.007	−23.9 ± 1.2	4.6 ± 1.0
NV-B_2_	163 ± 1	0.160 ± 0.013	25.5 ± 2.3	4.5 ± 1.1
NV-B_3_	152 ± 1	0.240 ± 0.025	−26.6 ± 1.8	3.6 ± 1.2
NV-B_4_	110 ± 8	0.244 ± 0.014	−27.1 ± 0.8	3.2 ± 0.7
NV-C	243 ± 1	0.181 ± 0.013	−29.5 ± 0.4	6.1 ± 1.0
NV-C_0.5_	575 ± 6	0.224 ± 0.017	−23.1 ± 0.5	4.8 ± 0.8
NV-C_1_	350.4 ± 4	0.182 ± 0.012	−23.3 ± 0.9	4.7 ± 0.5
NV-C_2_	318.9 ± 7	0.206 ± 0.013	−25.2 ± 0.6	4.4 ± 0.7
NV-C_3_	249.4 ± 4	0.164 ± 0.033	−22.4 ± 0.2	4.3 ± 0.6
NV-C_4_	232.9 ± 18	0.322 ± 0.065	−22.6 ± 1.7	3.8 ± 1.6
NV-D	204.1 ± 2	0.190 ± 0.005	−40.3± 1.6	10.0 ± 1.0
NV-D_0.5_	603 ± 10	0.388 ± 0.018	−33.6 ± 1.7	6.7 ± 1.4
NV-D_1_	264.7 ± 2	0.235 ± 0.008	−5.8 ± 0.05	6.8 ± 1.2
NV-D_2_	240.1 ± 2	0.199 ± 0.031	−10.3 ± 0.1	6.7 ± 1.1
NV-D_3_	210.4 ± 2	0.189 ± 0.014	−12.8 ± 1.5	4.3 ± 1.0
NV-D_4_	484.6 ± 16	0.671 ± 0.08	−10.7 ± 1.2	3.7 ± 1.7

^1^ PdI: polydispersity index; ^2^ ZP: zeta potential; ^3^ D.I.: deformability index.

## Data Availability

The data presented in this study are available from the corresponding author upon request. The data are not publicly available due to privacy.

## Supplementary Materials

The following are available online at https://www.mdpi.com/article/10.3390/jfb12040074/s1, Figure S1: Rutin external calibration curve obtained using a UV-Vis spectrophotometer (Perkin Elmer Lambda 35, Waltham, MA, USA). The figure reports representative data from three independent experiments; Figure S2: Cytotoxic effects of empty NV-A and DMSO, used for solubilizing rutin in the other in vitro experiments, of which the concentrations referred to A4 and the free rutin solution concentrations used at 24 h, 48 h, and 72 h. The results are expressed as the mean value of three independent experiments ± standard deviation.
