# Supplementary material for: Rutin-Loaded Nanovesicles for Improved Stability and Enhanced Topical Efficacy of Natural Compound
Source: J Funct Biomater. 2021 Dec 13;12(4):74. doi: 10.3390/jfb12040074 (PMC8704772; doi:10.3390/jfb12040074)
Supplement: Supplementary file 1 [file jfb-12-00074-s001.zip › jfb-1482802-supplementary.pdf]

Rutin-Loaded Nanovesicles for Improved Stability and Enhanced Topical Efficacy of Natural Compound

Maria Chiara Cristiano [1], Antonella Barone [1], Antonia Mancuso [2], Daniele Torella [1] and Donatella Paolino[1,*]

[1] Department of Experimental and Clinical Medicine, University "Magna Græcia" of Catanzaro, 88100 Catanzaro, Italy; mchiara.cristiano@unicz.it (M.C.C.); barone@unicz.it (A.B.); dtorella@unicz.it (D.T.)
[2] Department of Health Sciences, University of Catanzaro "Magna Graecia", 88100 Catanzaro, Italy; antonia.mancuso@unicz.it (A.M.)
* Correspondence: paolino@unicz.it; Tel.: +39-0961-369-4211

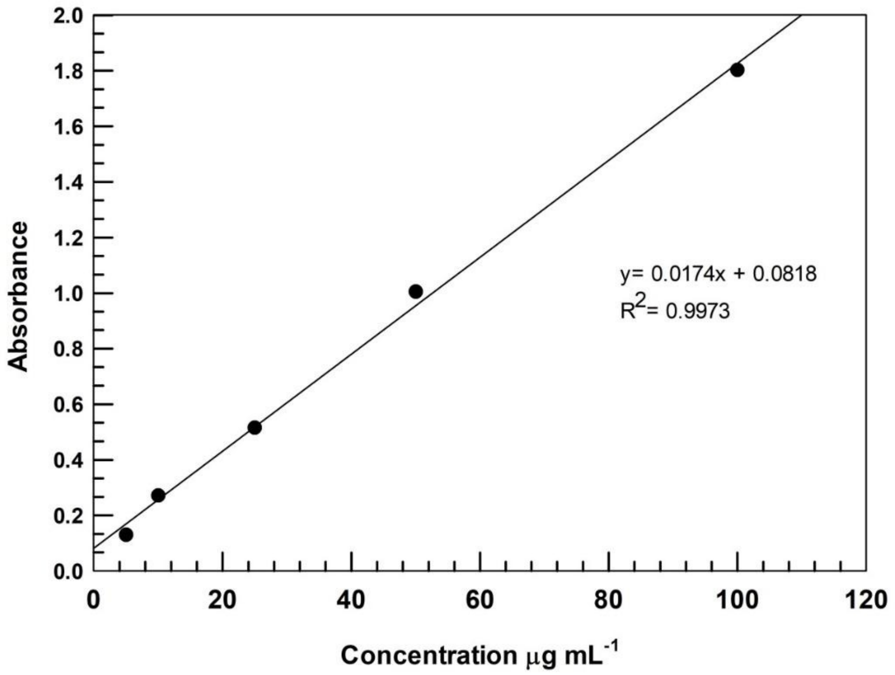

Figure S1. Rutin external calibration curve obtained using a UV-Vis spectrophotometer (Perkin Elmer Lambda 35, Waltham, Massachusetts, USA). The figure reports representative data from three independent experiments.

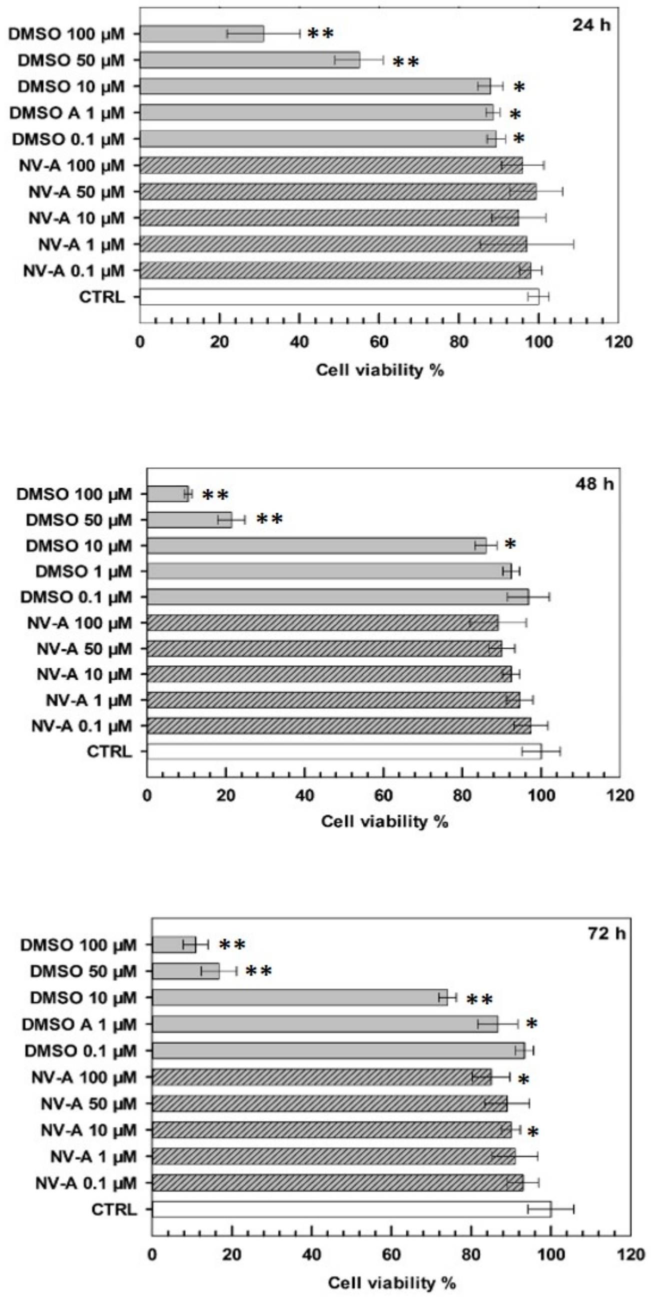

Figure S2. Cytotoxic effects of NV-A and DMSO, used for solubilizing rutin in the other in vitro experiments, of which the concentrations referred to NV-A_4 and the free rutin solution concentrations used at 24 h, 48 h, and 72 h. The results are expressed as mean value of three independent experiments ± standard deviation.
